# High-accuracy detection of supraspinatus fatty infiltration in shoulder MRI using convolutional neural network algorithms

**DOI:** 10.3389/fmed.2023.1070499

**Published:** 2023-05-25

**Authors:** Juan Pablo Saavedra, Guillermo Droppelmann, Nicolás García, Carlos Jorquera, Felipe Feijoo

**Affiliations:** ^1^School of Industrial Engineering, Pontificia Universidad Católica de Valparaíso, Valparaíso, Chile; ^2^Research Center on Medicine, Exercise, Sport and Health, MEDS Clinic, Santiago, Chile; ^3^Health Sciences PhD Program, Universidad Católica de Murcia UCAM, Murcia, Spain; ^4^Principles and Practice of Clinical Research (PPCR), Harvard T. H. Chan School of Public Health, Boston, MA, United States; ^5^Facultad de Ciencias, Escuela de Nutrición y Dietética, Universidad Mayor, Santiago, Chile

**Keywords:** classification, deep learning, fatty infiltration, MRI, supraspinatus

## Abstract

**Background:**

The supraspinatus muscle fatty infiltration (SMFI) is a crucial MRI shoulder finding to determine the patient’s prognosis. Clinicians have used the Goutallier classification to diagnose it. Deep learning algorithms have been demonstrated to have higher accuracy than traditional methods.

**Aim:**

To train convolutional neural network models to categorize the SMFI as a binary diagnosis based on Goutallier’s classification using shoulder MRIs.

**Methods:**

A retrospective study was performed. MRI and medical records from patients with SMFI diagnosis from January 1st, 2019, to September 20th, 2020, were selected. 900 T2-weighted, Y-view shoulder MRIs were evaluated. The supraspinatus fossa was automatically cropped using segmentation masks. A balancing technique was implemented. Five binary classification classes were developed into two as follows, A: 0, 1 v/s 3, 4; B: 0, 1 v/s 2, 3, 4; C: 0, 1 v/s 2; D: 0, 1, 2, v/s 3, 4; E: 2 v/s 3, 4. The VGG-19, ResNet-50, and Inception-v3 architectures were trained as backbone classifiers. An average of three 10-fold cross-validation processes were developed to evaluate model performance. AU-ROC, sensitivity, and specificity with 95% confidence intervals were used.

**Results:**

Overall, 606 shoulders MRIs were analyzed. The Goutallier distribution was presented as follows: 0 = 403; 1 = 114; 2 = 51; 3 = 24; 4 = 14. Case A, VGG-19 model demonstrated an AU-ROC of 0.991 ± 0.003 (accuracy, 0.973 ± 0.006; sensitivity, 0.947 ± 0.039; specificity, 0.975 ± 0.006). B, VGG-19, 0.961 ± 0.013 (0.925 ± 0.010; 0.847 ± 0.041; 0.939 ± 0.011). C, VGG-19, 0.935 ± 0.022 (0.900 ± 0.015; 0.750 ± 0.078; 0.914 ± 0.014). D, VGG-19, 0.977 ± 0.007 (0.942 ± 0.012; 0.925 ± 0.056; 0.942 ± 0.013). E, VGG-19, 0.861 ± 0.050 (0.779 ± 0.054; 0.706 ± 0.088; 0.831 ± 0.061).

**Conclusion:**

Convolutional neural network models demonstrated high accuracy in MRIs SMFI diagnosis.

## Introduction

Rotator cuff tears (RCTs) are among the most critical musculoskeletal conditions of the shoulder (1). This prevalence affects worldwide (2), resulting in direct and indirect economic burdens for patients and healthcare systems (3). Furthermore, this progressive degenerative condition (4) affects both sexes, and its incidence in the general population increases with age (5).

Image medical analysis plays a significant role in diagnosis and the optimal detection of the tear magnitude, allowing therapeutic planning resolutions, including physical therapy and surgical repair (6). Many imaging techniques have been developed for the detection of RCTs. Magnetic resonance imaging (MRI) presents the highest diagnostic value (sensitivity and specificity) for detecting any lesion (7, 8), especially for evaluating the integrity of the rotator cuff in tear size. Another essential radiological aspect of assessing the MRI shoulder is atrophy and fatty infiltration. Patients with a low stage of fatty infiltration have significantly better outcomes than those with a severe condition, since patients who present a re-tear are the most affected (9, 10).

For this reason, to determine the magnitude the SMFI, Goutallier et al. proposed a classification with five stages ranging from 0 to 4 (11). However, the original proposal has been adapted with MRI by Fuchs et al. (12) using three stages, combining stages zero and one as normal, two as moderate, and three with four as severe fatty infiltration. In the MRI adaptation of the classification, there has been controversy regarding the ideal technique for grading (13).

One of the most significant challenges in image diagnosis is reducing the variability between observers in assessing rotator cuff muscle quality on MRI (14). Recent studies have implemented the use of Artificial Intelligence (AI), Machine Learning (ML), and particularly Deep Learning (DL) techniques to improve the accuracy of diagnosis, helping radiologists with the interpretation of imaging data (15). This process has been facilitated by developing AI and ML tools and incorporating these into the diagnostic support of medical images (16). Also, as it is common to have small datasets in medical imaging, transfer learning using well-trained non-medical ImageNet datasets has shown promising results for medical image analysis in recent years. Some of the most used DL architectures in medical imaging analysis (17) include Inception-v3 (18), ResNet-50 (19), and VGG-19 (20).

Random forest (RF) and DL techniques, such as convolutional neural network (CNN), have been used to identify the segmentation of rotator cuff muscles on MRI (21). Also, automatic algorithms have been implemented to detect supraspinatus muscle atrophy (22), and detection of supraspinatus tears on MRI (23). However, such algorithms have not yet been implemented to detect this structure’s fatty infiltration level. Incorporating these artificial intelligence tools would improve diagnostic precision and patient prognosis. Kim (22) demonstrated CNNs’ ability to segment the supraspinatus muscle and supraspinatus fossa to calculate their ratio in an MRI dataset. Similarly, Ro and collaborators (24) developed a model that analyzes the muscle proportion in the supraspinatus fossa and quantifies fatty infiltration in MRI through Otsu thresholding (25). The Otsu thresholding is used to create pixel clusters from grayscale images and optimizes the pixel intensity value to establish foreground and background. In this case, the foreground would be fat, and the background would be muscle. This method is highly influenced by the difference in pixel intensity due to fatty infiltration level. This was addressed by computing a standard deviation for every Goutallier level. Using this method, Otsu thresholding showed 0.06; 4.68; 20.10; 42.86; and 55.76 for grades 0, 1, 2, 3, and 4, respectively. Finally, in the context of RCT and fatty infiltration imaging analysis, Taghizadeh (26) developed a convolutional neural network model to automatically quantify and characterize the degeneration of rotator cuff muscles from CT images. The backbone of this model is the U-Net architecture, which can segment muscle fossa into a pre-morbid state. Most convolutional neural network models have been used to segment regions of interest, including supraspinatus, infraspinatus, and subscapular muscles. Since Goutallier’s grade scale is a qualitative method and diagnoses are highly influenced by clinicians’ and experts’ intuitive judgment, literature has claimed that classification of Goutallier’s grade via DL methods is not an easy task (24).

To assess this hypothesis, this study aims to build a DL architecture to classify patients as “risky” or “not risky” based on the Goutallier’s supraspinatus fatty infiltration classification from shoulder MRI to help clinicians and medical staff in decision-making. Results demonstrate that DL models provide high accuracy and classification accuracy (discriminatory capacity) for Goutallier’s supraspinatus fatty infiltration levels.

## Materials and methods

### Study design

This study was designed as a retrospective and one site study. It was written following the Strengthening the Reporting of Observation studies in Epidemiology (STROBE) guideline. All patients record were obtained from a MRI exam at MEDS Clinic in Santiago, Región Metropolitana, Chile. This study started on September 25th, 2020.

### Datasets characteristics

The dataset used in this work comprises MRI and medical records from patients with an SMFI diagnosis who underwent examinations from January 1st, 2019, to September 20th, 2020. MRI images were saved in DICOM format, a widely used file format in medical imaging contexts. This format can save images, patient information, and study characteristics in one file. Each MRI image in the data set is obtained from a shoulder T2-weighted Y-view. The patient data were anonymized before being analyzed descriptively.

**Figure 1:**
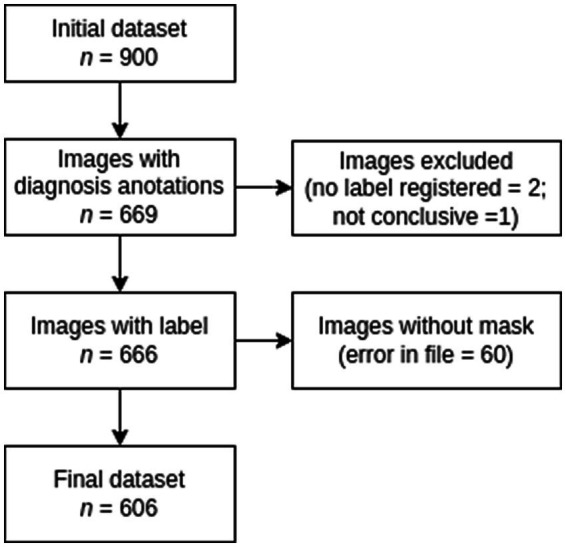
Flowchart for dataset selection.

The initial dataset contained 900 MRI studies. But 669 images had valid annotations. Then, a musculoskeletal radiologist labeled the images based on Goutallier’s fatty infiltration level. Two labeled images were excluded due to missing label records, and one was excluded because it was not conclusive for fatty infiltration analysis. After this process, 666 images were selected to perform manual segmentation. Sixty images had pixel configuration errors, and thus no segmentation could be done. The final dataset consists of 606 images, Figure 1.

**Figure 2:**
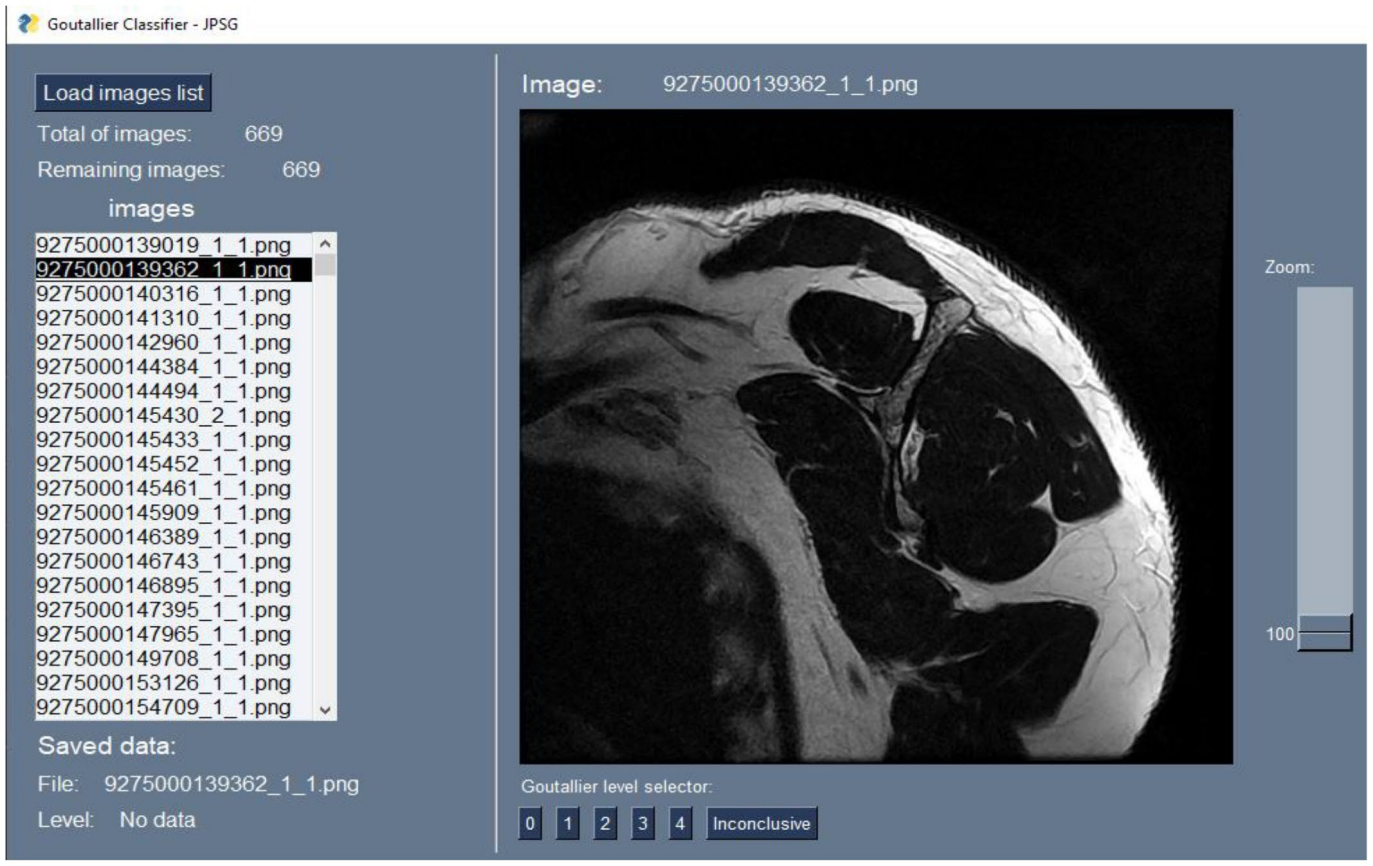
Custom software interface.

To perform the labeling process, we developed a simple Python software, Figure 2, that reads a folder with all the images to be annotated and then shows the MRI image one at a time. The radiologist selects the diagnosis for that MRI image. The program creates a two-field JSON file with the decision made for the professional for each image. One field is the image ID, and the other is the label record selected by the radiologist. These labels are our study’s ground truth.

### Statistical analysis

Dataset was analyzed and statistical tests were computed. For the analysis, python (with libraries such scipy) were used. Normality tests were performed. Statistical differences between groups were computed using the Mann–Whitney U test or t-test. A value of p of 0.05 was used to measure statistical significance. Descriptive analysis over the age of the patients was also performed and presented as mean and standard deviation (m ± sd). Percentages and frequencies are presented as statistical description for categorical.

Models’ performances were computed and compared using accuracy, sensitivity, specificity, and AU-ROC. A binary classifier outputs one of two possible values for a given input, 0 or 1. For every input there is an actual expected output, which is also 0 or 1. Table 1, also known as confusion matrix, shows the four possible outcome situations.

**Table 1:** Confusion matrix.

Classifier		Predicted	
		0	1
Actual	0	True Negative (TN)	False Positive (FP)
	1	False Negative (FN)	True Positive (TP)

We computed accuracy, sensitivity, specificity as follows:

- Accuracy: (TN + TP)/(TN + FP + FN + TP)
- Sensitivity (True positive rate): TP/(TP + FN)
- Specificity: TN/(TN + FP).

Area under the receiver operator curve or (AU-ROC) is a measure of the performance of the classifier regardless the threshold defined to translate probability scores to class decision. The horizontal axis corresponds to recall, or sensitivity, and the vertical axis corresponds to the precision, computed as TP/(TP + FP). As both axes are limited to 1, the maximum value of the area under the curve inside the square is 1, therefore, the closer to 1 the better the classifier. A random classifier will have an AU-ROC equal to 0.5.

In the case of the model performance, 95% confidence interval over the mean for the metrics, such as accuracy, sensitivity, specificity, and AU-ROC.

### Data preparation

The data preparation consisted of two main steps. First, the correct labeling of each image and the manual segmentation of the region of interest (ROI). All data in DICOM file format was processed with the MicroDICOM software to export images to PNG format. This allowed us to use fewer computational resources, as extracting images on the fly was unnecessary. Also, some Python libraries, such as PySimpleGUI, used to create the custom labeling software, only accept PNG format as input. We set the exported image resolution to the same as the original to avoid further mismatches between the image and its segmentation mask.

**Figure 3:**
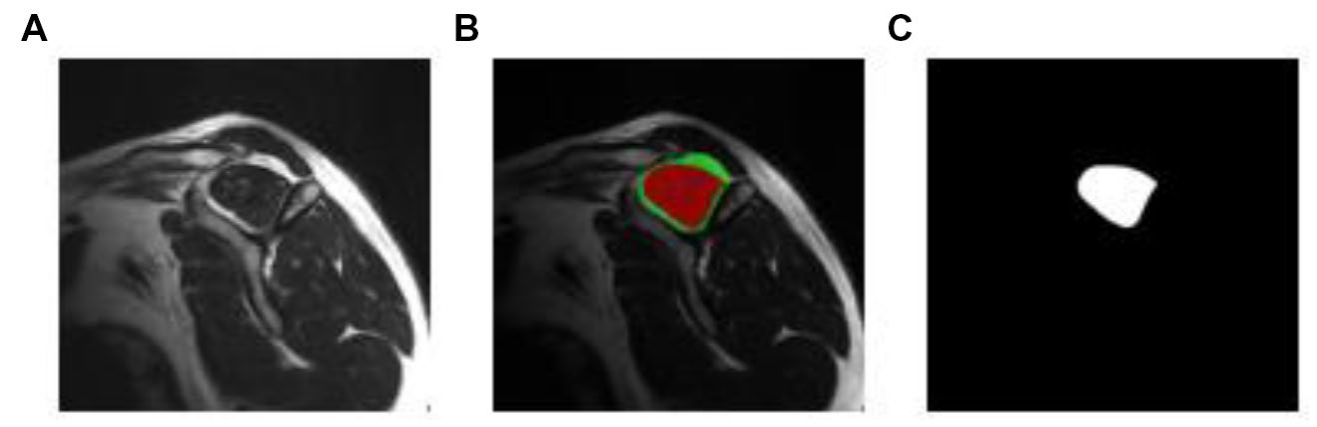
Manual segmentation process. Original, manual segmentation from ITK-Snap, and mask result, in figures **(A–C)**, respectively.

**Figure 4:**
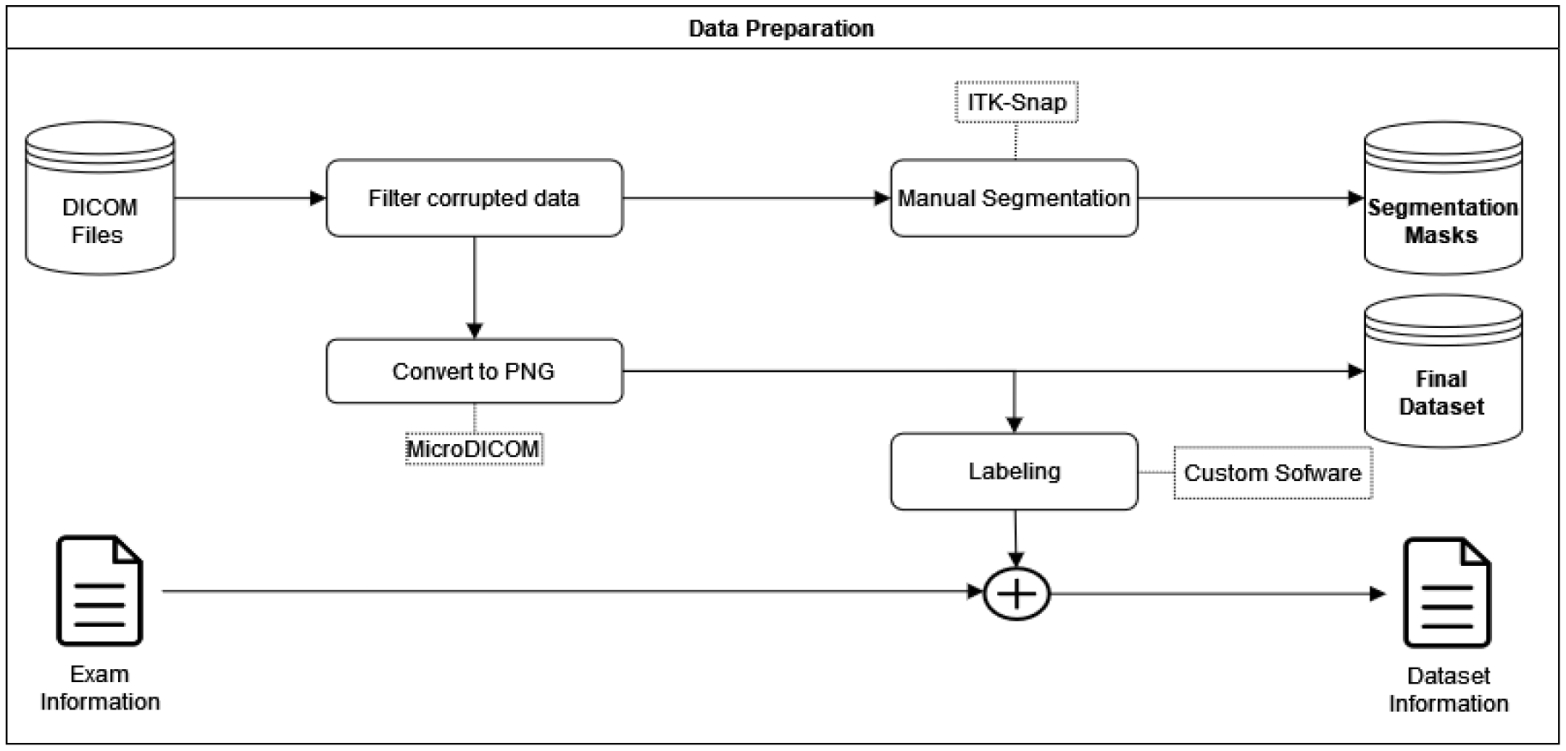
Data preparation.

Regarding the segmentation of the ROI, the original DICOM files were used to create manual segmentation (identify the ROI in each image). The segmented areas were the supraspinatus fossa and the supraspinatus muscle. Figure 3 shows a sample segmentation. Panel (a) displays the original image, panel (b) the manually created segmentation masks, and panel (c) the segmented area masks. Each MRI image was segmented using the ITK-Snap software (27). At the end of the data preparation process, we obtained the original MRI images in PNG format, the segmentation masks, and label information for every image. The data preparation workflow is shown in Figure 4.

### The definition and fatty infiltration criteria

**Figure 5:**
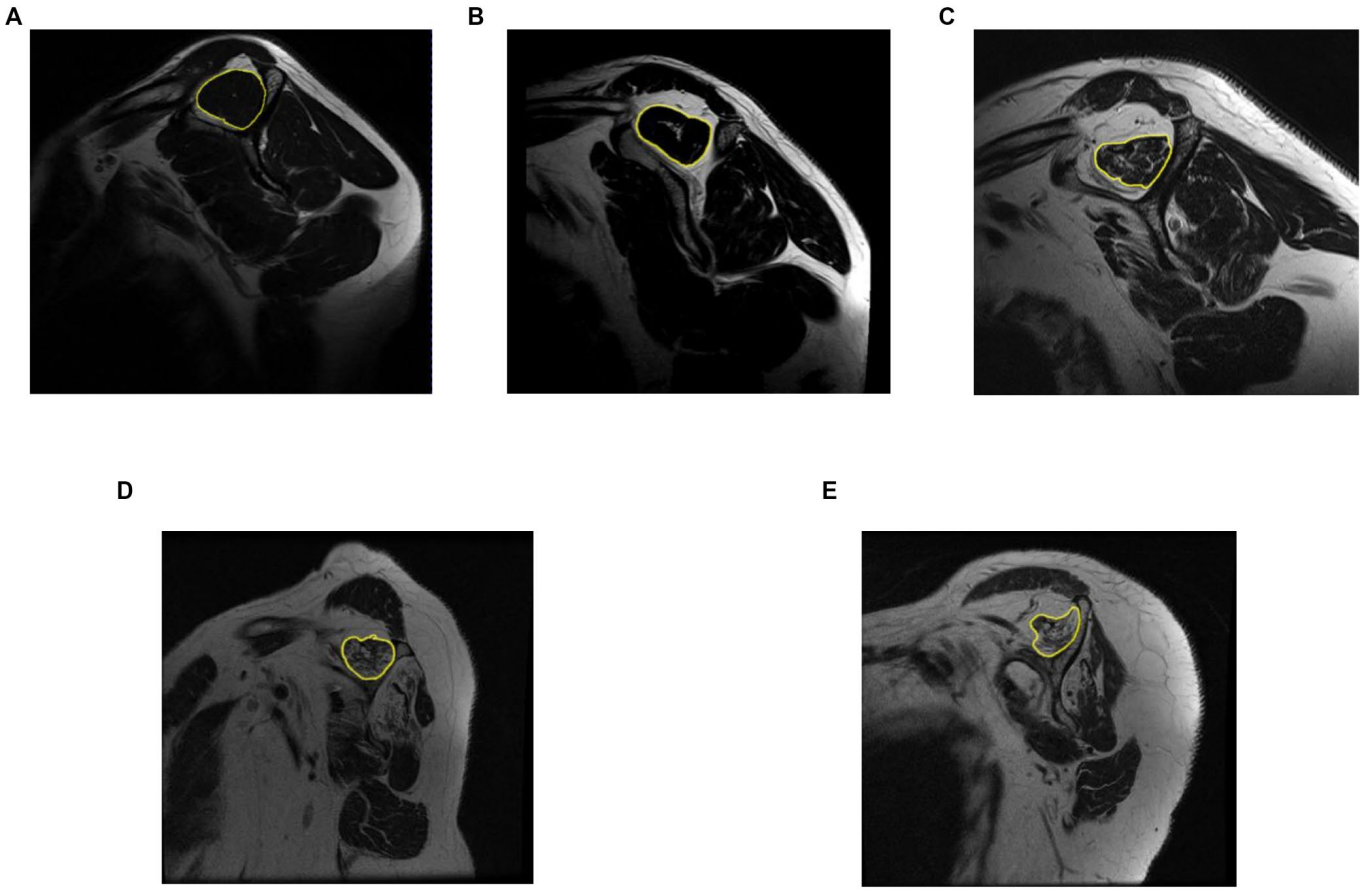
Representative MRI for each Goutallier’s fatty infiltration scale. Level 0,1, 2, 3, and 4, are shown in sub-image **(A–E)**, respectively.

We based our criteria on Goutallier’s fatty infiltration definitions. The original paper proposed five levels of fatty infiltration (zero to four) about the qualitative presence of fat in the muscle. A level of zero means there is no fat in the muscle. As fatty infiltration increases, Goutallier’s scale assigns a greater value. A level four means that there is more fat than muscle present. Figure 5 shows a representative MRI for every Goutallier’s fatty infiltration level.

As shown in Table 2, we studied DL techniques’ discriminatory (binary classification) power using five cases. In each case, we defined a positive and negative class composed of different Goutallier levels. Samples that belonged to the positive class were labeled as 1. Samples that belonged to the negative class were labeled as 0. The base case (case A) was used to assess the classification accuracy of no or low fatty infiltration (Goutallier 0 and 1) against high fatty infiltration (Goutallier 3 and 4). Goutallier level 2 is not considered in this case. This allowed us to assess whether the DL techniques can differentiate between no-fatty and high-fatty infiltration cases. Cases B to E is used as a sensitivity analysis of the classification capacity of the DL techniques.

**Table 2:** Class designation in every case for fatty infiltration levels.

Case	Fatty infiltration levels in		Set size	
	Negative class	Positive class	Negative class	Positive class
A	0, 1	3, 4	517	38
B	0, 1	2, 3, 4	517	89
C	0, 1	2	517	51
D	0, 1, 2	3, 4	568	38
E	2	3, 4	51	38

**Figure 6:**
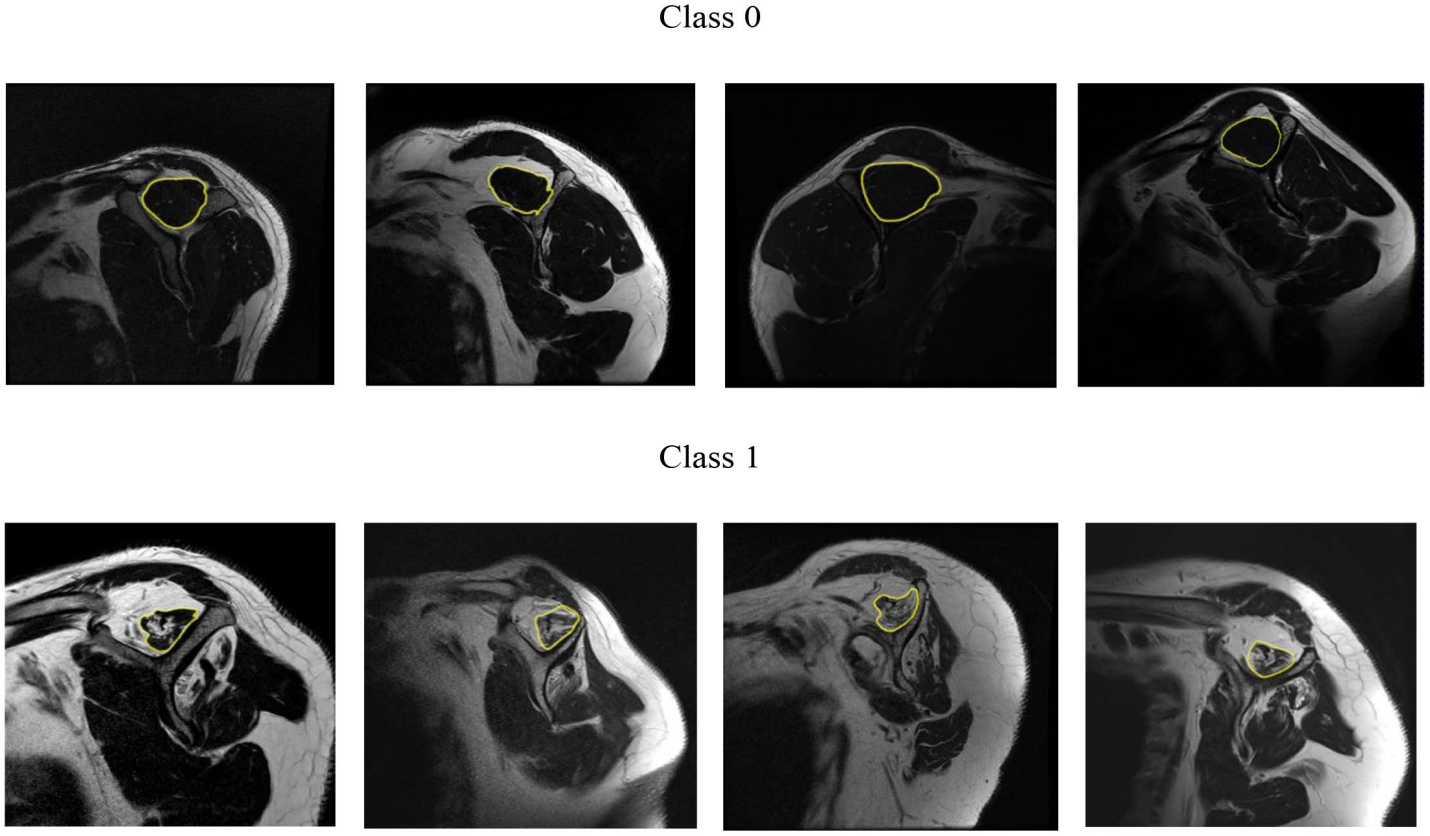
Random samples from class 0 and class 1 for the case A.

Based on the above definition of cases, a sample that belonged to class 1 (positive) was considered “risky.” A sample that belonged to class 0 (negative) was considered “no risky.” A few random samples from class 0 and class 1 are shown in Figure 6 for the case A. This classification is used since we aimed to help clinicians make decisions about proper treatment for patients based on the quality of the supraspinatus muscle. In every case, the positive and negative classes were different.

### Model development and training

**Figure 7:**
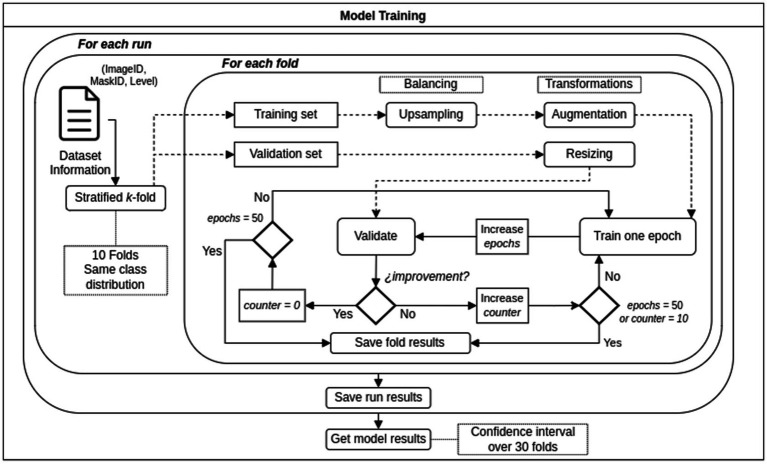
Training workflow.

**Figure 8:**
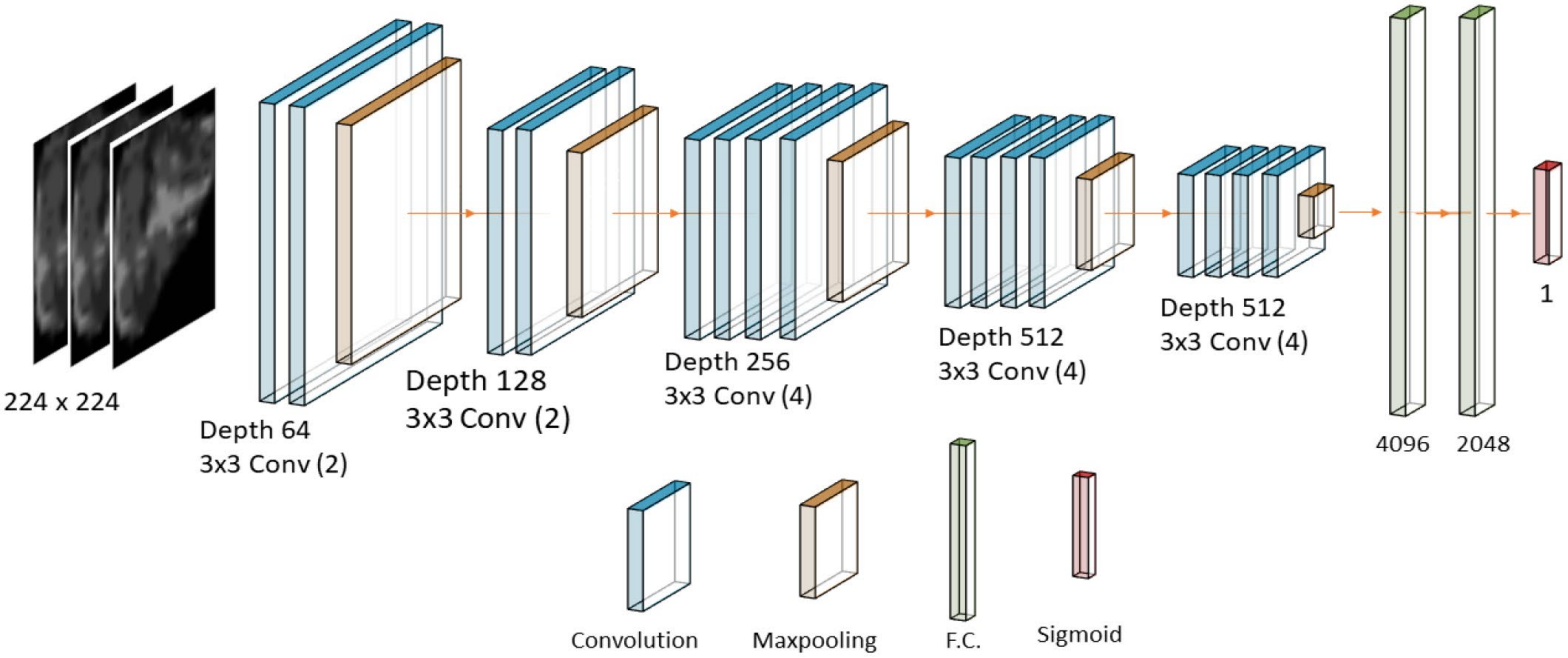
Diagram of the VGG/19 architecture.

Three models based on well-known architectures were trained: VGG-19, Inception-v3, and ResNet-50, and compared their performance in terms of classification accuracy. For every model, the learning rate and average time were processed. Figure 7 shows the general training workflow. In terms of the architecture, the convolutional layers for every model remained the same as in the original, and only the classifier was modified. We replaced the last layer of every model with a 1,000-unit wide and SoftMax activation function with a single neuron with a sigmoid activation function because our problem was binary classification. In the case of VGG-19, we also reduced the size of the most outer fully connected layer from 4,096 neurons to 2048, which helped to avoid overfitting, Figure 8. We used transfer learning from ImageNet weights to train the models. The backbone of the original architecture was used as a feature extractor, and its layers were frozen. Then, only the fully connected layer parameters were optimized. In addition, every model architecture was created to admit three-channel images (RGB) as input. We simulate an RGB image from a gray-scale MRI by copying the same channel two times. Then, the three versions of the same single channel were stacked into a three-channel image.

### Stratified k-fold cross validation

As we had a small dataset, stratified k-fold cross-validation was performed (28, 29). This method allowed us to use most of the data for training and reduce the impact of the data selection in the results as would happen in a 20/80 random split, for example. We choose k equals to 10 and thus, 10 subgroups from the original data were created. That the cross-validation process is stratified means that every subgroup maintains the same class distribution of the original dataset. In each of the 10 training runs nine groups were used for training and one group for validation. We repeat three times the complete process of creating the 10 subgroups and running the training process. The performance of the model is calculated as the average of 30 training runs, and the confidence intervals for each were also found. The training and validation process based on stratified k-fold cross-validation follows the methodology described in (28, 29) when models are trained using small datasets.

### Random data split

Additional to the assessment of the DL models using stratified K-fold cross validation, we evaluate the DL architectures using a new data set which has not been used during the training process. To do so, we trained the DL architectures using a random train/validation/test (70%/20%/10%, respectively) split. Downsampling of the majority class is performed over the training data only. The learning rate was set to 1e-06, 1e-04 and 1e-03 for VGG-19, Resnet50, and Inception V3, respectively. We train the model for 30 epochs and compute its accuracy, specificity, and sensitivity using the external new test data set (10% of the existing data) not used in training.

### Augmentation and data balancing techniques

The data was highly imbalanced. This could lead the model to learn better from the most represented class than the minority class or lead to a highly overfitted model. We performed a balancing technique on the minority class to avoid or minimize these problems. In every 10 cross-validation processes, we over-sample the minority class on the training set until both classes have approximately the same number of samples. The validation set in the K-fold cross validation process remains imbalanced to validate the model similar to the real-world collection of images. Data augmentation was also performed on every image from the training set that was fed to the model. Augmentation is accomplished by rotating any grade value in ±35° and horizontally flipping with a probability of 0.5.

### Training and optimization of hyper-parameters

All the DL models were trained using the Adam optimizer in standard configuration (weight decay = 0.9; beta = 0.999) for 50 epochs. The training process was stopped if there were no improvements in the last 10 epochs, and the best performance was saved. We only optimized the learning rate.

Before we fed the DL model with data, the region of interest was obtained from the segmentation mask for every image. This process is carried out automatically by the algorithm. It took the original image and the corresponding mask and cropped the region of interest. Then, only the ROI was fed to the DL models. The size of the input image was determined by the model’s architecture requirements, which are 224 [px] squared images for the VGG-19 and the ResNet-50 architectures, and 299 [px] squared images for the InceptionV3. The cropped image was resized to meet those requirements.

## Results

### Statistical analysis results

A total of 606 patients (55% were males) with 606 MRI with RCTs were included in our analysis. The patient’s average age was 55.1 ± 13.2 years. Data demonstrated the presence of all different Goutallier levels in imagological exams. An asymmetrical Goutallier distribution was found. More than 82% of the images belong to the 0 and 1 grades, showing an imbalance toward low fatty infiltration, as follows: Goutallier 0 (66.50%); Goutallier 1 (18.81%), Goutallier 2 (8.42%), Goutallier 3 (3.96%), and Goutallier 4 (2.31%). Also, the female group has more samples in higher grades than the male without statistical significance. The distribution of patient data is shown in Table 3.

**Table 3:** Quantity and proportions of sex by Goutallier’s level.

Goutallier Level	*N* (%)	Female		Male		Value of *p*	
		*N* (%)	Age mean (SD)	*N* (%)	Age mean (SD)	*N*	Age
0	403 (66.50)	140 (35)	53.06 (10.55)	263 (65)	49.24 (13.13)	0.477	***
1	114 (18.81)	74 (65)	61.50 (10.37)	40 (35)	63.58 (8.17)	0.465	0.371
2	51 (8.42)	31 (61)	66.65 (9.53)	20 (39)	66.40 (10.13)	0.447	0.992
3	24 (3.96)	16 (67)	68.88 (7.74)	8 (33)	64.25 (7.59)	0.424	0.230
4	14 (2.31)	13 (93)	67.31 (7.33)	1 (7)	N.A.	0.354	0.8
Total	606 (100)	274 (45)	58.47 (11.67)	332 (55)	52.42 (13.81)	0.483	

Mann–Whitney or *t*-test were used to compute the significance (alpha 0.05). ***, statistically significant

### Model performance

The learning rate used in every case and model and the average processing time were identified in Table 4. The shortest time was registered in the E case, using the Inception-v3 model with 0.34 ± 0.14 h. These results depend on the maximum number of epochs that the model runs until reaching its best validation loss, and thus, the training process is stopped and the training ends. In some cases, it is less than 50 epochs. In addition, the smaller the total size of the training set, the less time it takes to complete the training process. The E case has only 89 samples in total. On the other hand, the longest recorded time was registered in the VGG-19 model in the C case with 3.87 ± 0.35 h.

**Table 4:** Learning rate and average processing time (C.I. 95%) for every case and model.

Case	Model	Learning rate	Processing time	Max. epochs
A	VGG-19	10^−6^	3.51 ± 0.20	31.6 ± 3
	ResNet-50	10^−4^	2.35 ± 0.22	20.7 ± 3.3
	Inception-v3	10^−3^	1.55 ± 0.14	11.8 ± 2.5
B	VGG-19	10^−6^	3.83 ± 0.27	33.1 ± 2.5
	ResNet-50	10^−3^	1.12 ± 0.34	6.8 ± 2.2
	Inception-v3	10^−3^	1.40 ± 0.19	9.3 ± 2.3
C	VGG-19	10^−6^	3.87 ± 0.35	33.3 ± 2.9
	ResNet-50	10^−5^	2.91 ± 0.22	26.9 ± 2.8
	Inception-v3	10^−4^	2.98 ± 0.53	22.3 ± 2.9
D	VGG-19	10^−6^	3.20 ± 0.25	27 ± 3.2
	ResNet-50	10^−5^	3.09 ± 0.46	25.9 ± 2.9
	Inception-v3	10^−3^	1.40 ± 0.01	8.8 ± 2.0
E	VGG-19	10^−3^	0.42 ± 0.03	11.6 ± 3.0
	ResNet-50	10^−4^	0.86 ± 0.20	22.5 ± 3.6
	Inception-v3	10^−4^	0.34 ± 0.14	9.4 ± 4.0

The DL architectures demonstrated outstanding performance using a shoulder MRI dataset. With a 10-fold cross-validation process, data was randomly divided into 10 non-overlapping folds. Nine folds were used as training sets and one as a validation set. The process was repeated three times; thus, three runs were obtained. This led to an average of 30 training loops.

**Figure 9:**
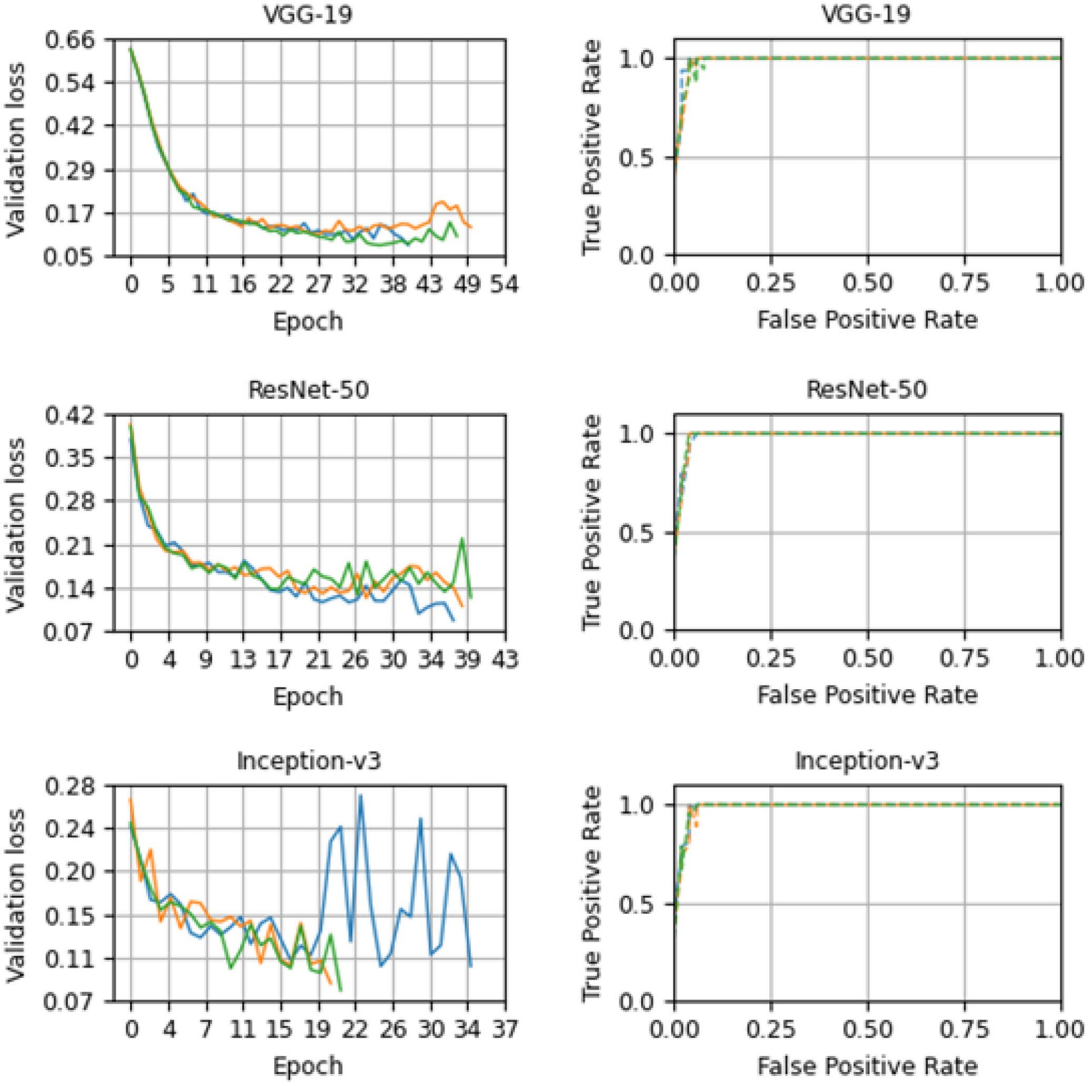
Loss and receiver operator curve plots for VGG-19, ResNet-50, and Inception-v3 models for base case (A). The results for the first, second, and third run are in color green, orange and blue, respectively.

Figure 9 shows the validation loss and AU-ROC curves for every model at every run. The three architectures show a decreasing validation loss at every epoch. At the beginning of the training process, the VGG-19 loss validation starts at 0.739 ± 0.006, 0.632 ± 0.007, and 0.631 ± 0.005 in the first, second, and third runs, respectively. Then, in the end, the validation loss was reduced to 0.225 ± 0.0053. In the case of Inception-v3, there is noticeably different behavior in one of the runs. This up-and-down loss value for the validation set could probably be explained due to the randomness in the process and the fact that the model could find a local minimum near the end. In any case, the last epoch showed an improvement in the validation loss value, and thus, it was recorded. Table 5 shows the starting value for the validation loss for every model. The model was run for a maximum of 50 epochs. We track the evolution of the loss function value. If the loss function did not decrease during 10 epochs, then the training process was terminated, and the results were computed.

**Table 5:** Confidence intervals (95%) for the starting validation loss in each run.

Model	Run 1	Run 2	Run 3
VGG-19	0.739 ± 0.006	0.632 ± 0.007	0.631 ± 0.005
ResNet-50	0.379 ± 0.024	0.403 ± 0.031	0.400 ± 0.047
Inception-v3	0.239 ± 0.050	0.265 ± 0.037	0.243 ± 0.068

The results confirm an optimized loss function. The loss function converges to zero as the learning progresses in the validation processes.

**Figure 10:**
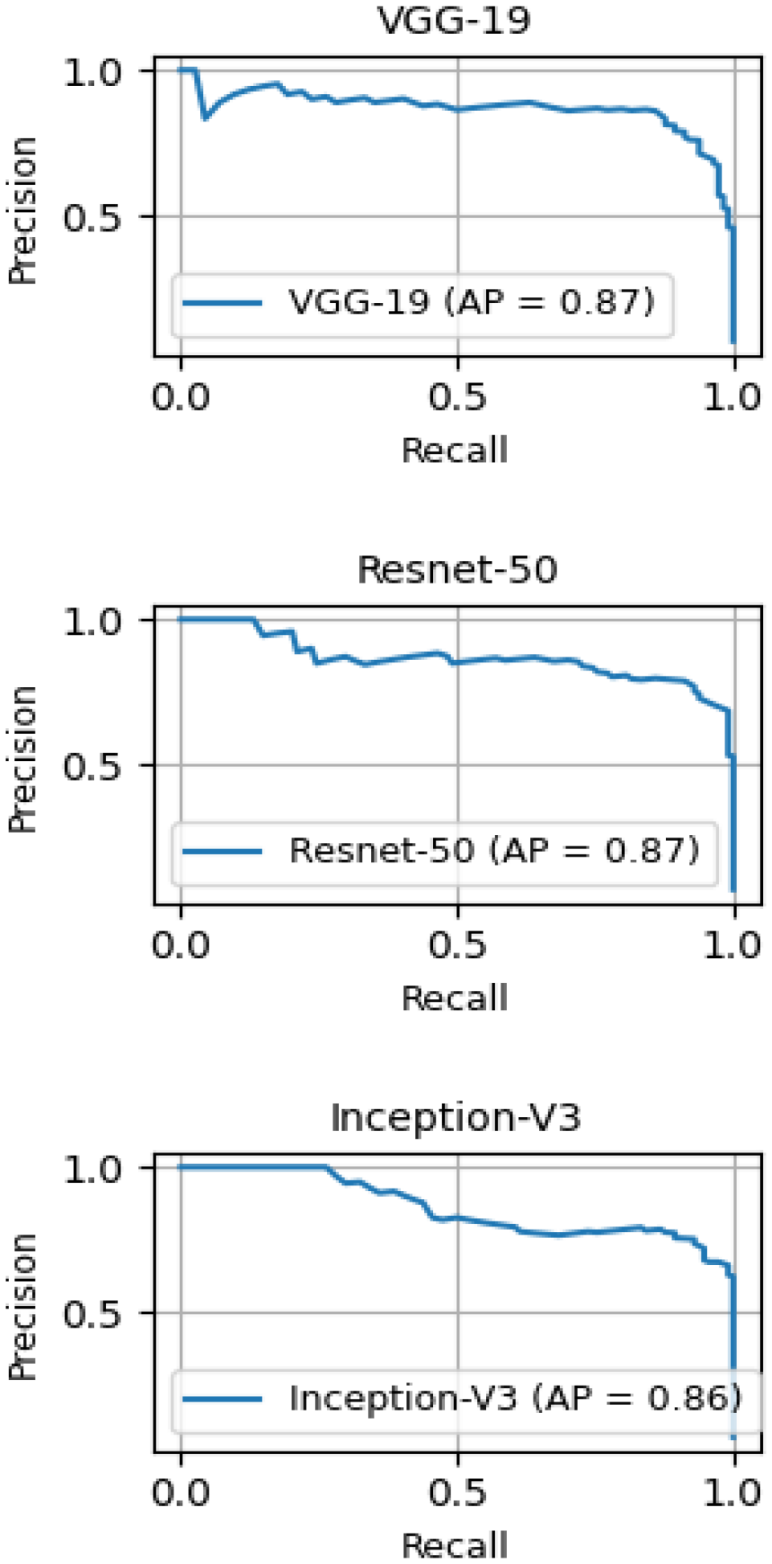
Mean Precision-recall curves for VGG-19, ResNet-50, and Inception-V3 models for the base case (A).

The model returns a value between 0 and 1, corresponding to the likelihood that the image belongs to the positive class. The value is then converted to binary based on a threshold. As the threshold value in our study, we utilized 0.5. The class will be considered positive if the model outputs a value greater than that. In contrast, if the model outputs a value lower than that threshold, the decision will be categorized as negative. One can compute the false positive and true positive rates under thresholds. The ROC curves in Figure 9 demonstrate the high performance of the models for various threshold values. The closer the curve is to (0.0, 1.0), the better the performance. To quantify curves, the area under the ROC curve was used. For our case A, VGG-19, ResNet-50, and Inception-v3 achieved 0.991 ± 0.003, 0.992 ± 0.003, and 0.991 ± 0.004, respectively for the area under the ROC curve (AU-ROC). Also, as shown in Figure 10, VGG-19 and ResNet-50 models showed the better performance when comparing precision-recall curves. When analyzing the per class prediction, the three models showed better performance in the negative class than in the positive class, which has fewer samples. Table 6 shows the confusion matrix for each model.

**Table 6:** Confusion matrix of VGG-19, Resnet-50, and Inception-V3 models for the case A validation set.

VGG-19		Predicted	
		0	1
Actual	0	1,512	39
	1	6	108

Resnet-50		Predicted	
		0	1
Actual	0	1,520	31
	1	9	105

Inception-V3		Predicted	
		0	1
Actual	0	1,522	29
	1	15	99

### Subgroup analysis

A subgroup analysis was developed to determine the best combination of binary classes for Goutallier fatty infiltration level detection. Accuracy, sensitivity, specificity, AU-ROC, and loss performance for every single convolutional neural network model after three runs of 10 training cycles each are shown in Table 7. The reported metrics values shown are based on the results obtained from the repeated cross validation process. The process allowed us to have several validation groups and hence estimate the mean and the confidence level of each model in every experiment. Since DL models tend to learn the training data well, we do not report the training accuracy. Instead, we provide the evolution of the Loss Function, which depicts how the training error (learning process of the model) evolves. We also clarify this in the revised manuscript.

**Table 7:** Mean train loss, validation loss, accuracy, sensitivity, specificity, and AU-ROC for every case and model (C.I. 95%).

Case	Model	Train loss	Validation loss	Accuracy	Sensitivity	Specificity	AU-ROC
A	VGG-19	0.225 ± 0.053	0.096 ± 0.010	0.973 ± 0.006	0.947 ± 0.039	0.975 ± 0.006	0.991 ± 0.003
	ResNet-50	0.394 ± 0.099	0.123 ± 0.011	0.976 ± 0.006	0.925 ± 0.053	0.980 ± 0.006	0.992 ± 0.003
	Inception-v3	0.474 ± 0.154	0.102 ± 0.009	0.974 ± 0.007	0.869 ± 0.085	0.981 ± 0.006	0.991 ± 0.004
B	VGG-19	0.345 ± 0.045	0.246 ± 0.014	0.925 ± 0.010	0.847 ± 0.041	0.939 ± 0.011	0.961 ± 0.013
	ResNet-50	0.563 ± 0.184	0.187 ± 0.022	0.936 ± 0.012	0.779 ± 0.057	0.963 ± 0.009	0.948 ± 0.017
	Inception-v3	0.332 ± 0.094	0.214 ± 0.012	0.933 ± 0.010	0.802 ± 0.039	0.956 ± 0.008	0.951 ± 0.013
C	VGG-19	0.453 ± 0.057	0.310 ± 0.016	0.900 ± 0.015	0.750 ± 0.078	0.914 ± 0.014	0.935 ± 0.022
	ResNet-50	0.605 ± 0.037	0.507 ± 0.008	0.896 ± 0.015	0.756 ± 0.079	0.909 ± 0.015	0.913 ± 0.025
	Inception-v3	0.587 ± 0.048	0.372 ± 0.013	0.914 ± 0.011	0.659 ± 0.056	0.939 ± 0.012	0.912 ± 0.019
D	VGG-19	0.299 ± 0.056	0.153 ± 0.018	0.942 ± 0.012	0.925 ± 0.056	0.942 ± 0.013	0.977 ± 0.007
	ResNet-50	0.631 ± 0.040	0.405 ± 0.010	0.928 ± 0.013	0.872 ± 0.066	0.932 ± 0.012	0.964 ± 0.012
	Inception-v3	0.494 ± 0.168	0.150 ± 0.011	0.941 ± 0.011	0.808 ± 0.078	0.950 ± 0.010	0.975 ± 0.007
E	VGG-19	0.519 ± 0.242	0.505 ± 0.138	0.779 ± 0.054	0.706 ± 0.088	0.831 ± 0.061	0.861 ± 0.050
	ResNet-50	0.664 ± 0.016	0.631 ± 0.012	0.700 ± 0.038	0.611 ± 0.102	0.756 ± 0.056	0.785 ± 0.053
	Inception-v3	0.696 ± 0.028	0.665 ± 0.008	0.678 ± 0.057	0.550 ± 0.103	0.766 ± 0.088	0.722 ± 0.072

Excellent performance for the three architectures in every case was demonstrated. In three out of four cases, all model configurations had AU-ROC values higher than 0.91 on average and thus performed well when classifying fatty infiltration levels. In the base case, the models got an AU-ROC mean value of over 0.99, the highest among the cases. Here, models had to separate lower to no fatty infiltration images from high to extreme fatty infiltration levels, which were very dissimilar. In addition, sensitivity and specificity for this case are more homogeneous among models. This means that the models perform well when classifying negative and positive samples as the false positive rate and true positive rate are over 0.92, except for Inception-v3, which has a lower value for sensitivity. On the other hand, the same architecture showed a higher specificity, with a mean value of 0.981.

### Random split performance

We also trained the model using a random train/validation/test split (training size: 413, validation size: 104, testing size: 58). Only the training data was down-sampled in order to account for unbalanced labels. As shown in Table 8, all models showed similar performance in the final testing data split (10% of the data) as the observed in the stratified k-fold cross-validation method, reaching, for instance for the VGG-19 model, 0.931, 1.0, and 0.925 for the accuracy, sensitivity, and specificity, respectively. This demonstrates the usability of DL techniques and that the models are not likely to be overfitted as demonstrated in the stratified k-fold process and in the 10% final random data split process. During the process of reviewing this paper, we were able to collect 20 more images. We added those images to the previous dataset and performed a random split experiment. We computed the performance of every model using this new dataset.

**Table 8:** Accuracy, sensitivity, and specificity for case A and all DL models using a random training/validation/test data split.

	Accuracy	Sensitivity	Specificity
VGG-19 (Case A)	0.931	1.0	0.925
ResNET50 (Case A)	0.948	0.8	0.962
Inception V3 (Case A)	0.965	0.8	0.981

## Discussion

This research is one of the first to demonstrate the capabilities of the DL models to classify SMFI in patients with RC conditions. The imagenological analysis considered an extensive novel shoulder T2-weighted MRI (30). This retrospective analysis applied various DL models, including the VGG-19, ResNet-50, and Inception-v3 architectures.

All diagnostics metrics demonstrated excellent results, achieving a high binary classification performance in every class of the Goutallier level. Distinctly high accuracy, sensitivity, and specificity among different architectures belonging to neural networks were found, specifically when the diagnosis was based on case A, that is, the negative class (Goutallier 0 or 1) and the positive class (Goutallier 3 or 4).

Traditionally, the scapular Y-view of the MRI, particularly the lateral-most T1 sagittal, is the most reliable indicator of the supraspinatus muscle status and is used for identifying FI (31). However, current standard shoulder protocols include sagittal oblique T2-weighted sequences to evaluate these findings (32). Despite that, recent data support ML methods’ crucial function in identifying various structures in medical images (33). For this reason, we proposed evaluating the most extensive collections of T2 MRI sequences.

The approach we described allows a practical solution when the grading system of FI is presented, reducing diagnostic uncertainty. Other experiences using artificial intelligence have been published. We highlight the exciting work Ro et al. (24) carried out. They implemented a novel model using only 250 patients (all of whom were diagnosed with atrophy and fatty infiltration of the supraspinatus muscle) to analyze the occupation ratio using a DL framework. They calculated the amount of FI in the supraspinatus muscle using an automated region-based Otsu thresholding technique. Their method allows segmenting the supraspinatus muscle and fossa, which lets them figure out the occupation ratio without automatically classifying the Goutallier level.

In our case, results demonstrated that artificial intelligence tools, particularly the VGG-19 architecture, can be used to support shoulder MRI diagnosis. Few studies in the musculoskeletal radiology literature have addressed the evaluation of RC muscles using these methods (34). Even though supervised deep learning with CNNs has been highly successful in medical imaging, particularly in MRI (35). However, based on the CNN tool, different studies have determined the need to count with more analysis to detect the supraspinatus muscle’s fatty infiltration (22).

Also, we identified some limitations. Firstly, our results used a binary classification method, even though the classification proposed by Goutallier presents five types of fatty infiltration. However, the binary performance showed great classification results, with an AUC of 0.991 [95% CI, ± 0.003] for the low to nonfatty infiltration against severe to extreme fatty infiltration (VGG-19 model). Therefore, a Fuch-type classification (12) could be more accessible to learn than a Goutallier-type classification. For this reason, it is necessary to have future studies that use multilabel classification methods. In addition, since the number of samples (images) in the data set was small, a training and validation set were created for the cross-validation process, however. The training and validation process used in this study follows related papers which faced similar data limitations (22, 24). To further assess the model performance, we used a training/validation/test random data split using 70%/20%/10% (train size: 413 validation size: 104, testing size: 58) for training, testing and validation, respectively. This allowed us to further confirm the good model performance in predicting class 0 and 1. In the future, more data is needed to further test the proposed models.

On the other hand, when we included category two (Goutallier type 2), the analysis reduced the capability to classify correctly. However, better performance was achieved when the type two class was added to the negative class. As in other publications, the present study was an image analysis; clinical factors and the patient’s history were not considered (24). Another essential point is that using these AI tools requires teamwork between clinical practitioners and engineering. Interdisciplinary work is necessary to improve people’s health.

In conclusion, CNN models, particularly VGG-19, showed outstanding performance in classifying SMFI using shoulder T2-weighted MRI in patients with RC conditions. AI models could be used to support the radiological diagnosis.

## Data availability statement

The raw data supporting the conclusions of this article will be made available by the authors, without undue reservation.

## Ethics statement

The studies involving human participants were reviewed and approved by Comité de Ética Científico Adulto del Servicio Metropolitano Oriente de la ciudad de Santiago de Chile (SSMO). Written informed consent for participation was not required for this study in accordance with the national legislation and the institutional requirements.

## Author contributions

JS: conceptualization, data curation, formal analysis, investigation, methodology, model development, training, and writing original draft. GD: data curation, formal analysis, investigation, validation, and writing original draft. NG: validation and review. CJ: resources and validation. FF: conceptualization, formal analysis, investigation, supervision, review, and editing. All authors contributed to the article and approved the submitted version.

## Funding

The authors received no financial support for the research and authorship. The publication was financially supported by the Universidad Mayor.

## Conflict of interest

The authors declare that the research was conducted in the absence of any commercial or financial relationships that could be construed as a potential conflict of interest.

## Publisher’s note

All claims expressed in this article are solely those of the authors and do not necessarily represent those of their affiliated organizations, or those of the publisher, the editors and the reviewers. Any product that may be evaluated in this article, or claim that may be made by its manufacturer, is not guaranteed or endorsed by the publisher.
